# *Global Health Action*: surviving infancy and taking first steps – the window is open, new challenges for existing niche may enlighten global health

**DOI:** 10.3402/gha.v7.23123

**Published:** 2014-01-31

**Authors:** Kenzo Takahashi, Jun Kobayashi, Kazuhiro Kakimoto, Yasuhide Nakamura

**Affiliations:** 1 Department of Epidemiology and Public Health, Graduate School of Medicine, Yokohama City University, Yokohama, Japan. kt_intl_@ja2.so-net.ne.jp or kenzo_gh@yokohama-cu.ac.jp; 2 Department of Global Health, School of Health Sciences, University of the Ryukyus, Okinawa, Japan; 3 Department of International Assistance, College of Human Sciences and Health, Osaka Prefecture University, Osaka, Japan; 4 Department of International Collaboration, Graduate School of Human Sciences, Osaka University, Osaka, Japan

With reference to the editorial ‘*Global Health Action*: surviving infancy and taking first steps’, we commend the success of the innovations in this newly established open-access global health journal, which sets a challenge to the world of scholars who deal with global health as an academic topic (1). The strategies of publishing Capacity Building and Study Design papers, as well as PhD Reviews, and providing mentorship have, in our opinion, not only filled the niches but have also bridged the 10/90 gap in health research that exists in global health (2, 3).

We welcome the Capacity Building article approach, as many overseas development assistance projects emphasize the importance of the process rather than the outcome, in order to ensure sustainability. Much time is required to foster mutual trust, achieve consensus, and plan a viable program in partner countries. Important lessons are learned throughout this process, and we strongly believe that sharing such lessons will help ensure the sustainability of both policy dialogs and the development which the project has nurtured (4). However, scientific journals on global health, other than *Global Health Action*, stress on a results-based approach and provide no opportunity to include these important lessons. We also expect the Study Design articles and PhD Reviews to provide a rich reservoir of ideas and advice and, as such, to provide a valuable knowledge base. In Japan, some of the young researchers coming from low- and middle-income countries are bureaucrats with access to national data, and their work has the potential to yield high-quality research evidence. They can certainly benefit from the mentorship provided by *Global Health Action*.

Finally, we support *Global Health Action's* concept of publishing article translations as supplementary online material, as this provides an avenue for getting research in other languages into the published literature. For example, in Japan, we have valuable experience in research and discussion on global health topics; unfortunately, such experience is documented in the Japanese language, making it impossible to share our discussion globally. This is no doubt the situation with other languages. Thus, we propose that *Global Health Action* should invite editors familiar with languages other than English. The invited editors could have three roles: 1) Organize special supplementary issues that deal with hot topics discussed in their respective countries. The editors could invite the contributions of several authors from these countries, write editorials that reference articles in other languages, and perform the role of contributing editor (as is already done in *Global Health Action*) (5). 2) Introduce a curated selection of English abstracts of articles on themes that may attract international readers. 3) Check for plagiarism in the original-language article. We expect that editors who perform the aforementioned functions would make *Global Health Action* a very global journal.

*Kenzo Takahashi* Department of Epidemiology and Public Health
Graduate School of Medicine
Yokohama City University, Yokohama, Japan kt_intl_@ja2.so-net.ne.jp or kenzo_gh@yokohama-cu.ac.jp *Jun Kobayashi* Department of Global Health, School of Health Sciences
University of the Ryukyus, Okinawa, Japan *Kazuhiro Kakimoto* Department of International Assistance
College of Human Sciences and Health Osaka Prefecture University, Osaka, Japan *Yasuhide Nakamura* Department of International Collaboration
Graduate School of Human Sciences, Osaka University Osaka, Japan

